# Distribution, Sources, and Water Quality Assessment of Dissolved Heavy Metals in the Jiulongjiang River Water, Southeast China

**DOI:** 10.3390/ijerph15122752

**Published:** 2018-12-05

**Authors:** Bin Liang, Guilin Han, Man Liu, Kunhua Yang, Xiaoqiang Li, Jinke Liu

**Affiliations:** School of Scientific Research, China University of Geosciences (Beijing), Beijing 100083, China; liangbin@cugb.edu.cn (B.L.); lman@cugb.edu.cn (M.L.); kunhuayang@cugb.edu.cn (K.Y.); xiaoqli@cugb.edu.cn (X.L.); liujinke@cugb.edu.cn (J.L.)

**Keywords:** dissolved heavy metals, the Jiulongjiang River, correlation analysis, factor and principal component analysis, water quality index, health risk assessment

## Abstract

In this study, the concentration of eight dissolved heavy metals (Ti, Cr, Mn, Fe, Ni, Mo, Sb, and Ba) in 42 water samples from the Jiulongjiang River, southeast China, were determined by inductively coupled plasma mass spectrometry (ICP-MS). Multivariate statistical methods, including correlation analysis (CA) and factor and principal component analysis (FA/PCA), were analyzed to identify the sources of the elements. Water quality index (WQI) and health risk assessment, including hazard quotient (HQ) and hazard index (HI), were used to evaluate water quality and the impacts on human health. Our results were compared with the drinking water guidelines reported by China, the World Health Organization (WHO), and the United States Environmental Protection Agency (US EPA), revealing that Ti, Mn, and Sb were not within approved limits at some sites and might be the main pollutants in the drainage basin. Based on the spatial distributions, Ti, Mn, Fe, Ni, and Mo showed good similarity, indicating that they might come from similar sources along the river. The CA results also showed that Ti, Mn, Fe, Ni, and Mo had a high correlation coefficient. The FA/PCA results identified three principal components (PC) that accounted for 79.46% of the total variance. PC 1 suggested that a mixed lithogenic and urban land source contributed to Ti, Mn, Fe, Ni, and Mo; PC 2 showed that Cr, Ni, and Mo were influenced by the discharge of industrial effluents; Sb had a strong loading on PC 3, which was controlled by mining activities. The results of the WQI indicated that the water in the Jiulongjiang River was basically categorized as excellent water, but the water quality levels in site W5 and N4 were poorer due to urban land use. Hazard quotient and HI values showed that Sb was a potential threat to human health, indicating that preventive actions should be considered in regard to mining activities in the upper reaches of Beixi stream.

## 1. Introduction

Globally, heavy metals are one of the most hazardous pollutants in water [1,2,3,4,5,6], and can be observed in dissolved phase [7,8,9,10], suspended particle phase [11,12,13], and sedimentary phases [14,15] in water systems. Dissolved heavy metals are generally more toxic than other phases [7,16]. Many heavy metal elements, such as Ti, Cr, Mn, Fe, Ni, Cu, Zn, Mo, Cd, Sb, Ba, Pb, etc., used to be detected in dissolved form due to industrial and domestic discharge without treatments [17,18,19]. Due to the toxicity, non-degradation, and bio-accumulation of heavy metals, an overbalance of concentrations can make water unsuitable for drinking and even cause severe risks to human bodies [4,20,21,22]. Statistically, about 80% of human disease is caused by water pollution, according to the World Health Organization (WHO) [23]. Therefore, continued research on dissolved heavy metals may be key in order to shed further light on some of the effects of pollution. Moreover, evaluating the effects of dissolved heavy metals on human health and water systems is considered as one of the most direct and important ways in which to judge the pollution present in water [1,10,23,24,25]. Thus, it is of great significance to understand the concentration, distribution, and sources of dissolved heavy metals, to assess water quality and health risks, to control water pollution, and to protect water resources [25].

Dissolved heavy metals can be released into the aqueous system by natural processes, such as bedrock weathering or volcanism [26,27], and element concentrations can be largely increased through anthropogenic factors, such as industrial wastes, sewage discharge, coal combustion, mining, and vehicle transportation [24,28,29]. Unfortunately, it is almost inevitable that water quality will be degraded with the rapid development of urban, industrial, and agricultural activities in a city. The Jiulongjiang River is the second longest river in Fujian Province, southeast China, and is also the source of irrigation and drinking water for many riverside towns [30]. In recent years, the drainage basin has received considerable attention in regard to its environmental problems, such as: (i) the discharge of industrial and domestic sewage [31]; (ii) the effects of local mining activities, given that mining and coal resources are widely distributed in the upper reaches of Beixi stream [32]; (iii) the inputs of chemical fertilizers and nutrients [32]; (iv) the extensive development of hydro power plants in the drainage basin [8,33]; (v) the effects of a large-scale livestock operation on the bank of the river [34], etc. These situations may have contributed to the increasing metal pollution of the Jiulongjiang River, and appropriate measures must be taken in order to ensure the protection of local human and environmental health. However, information on source identification and assessment of water quality has rarely been discussed in previous studies.

In this study, a geochemical survey on eight selected elements (Ti, Cr, Mn, Fe, Ni, Mo, Sb, and Ba) in 42 water samples has been conducted with the objectives to (i) investigate the spatial distribution characteristics of dissolved heavy metals in the river water; (ii) identify the sources of dissolved heavy metals with multivariate statistical methods, including correlation analysis (CA) and factor and principal component analysis (FA/PCA); (iii) evaluate water quality and distinguish the hazard impacts on human health by calculating the water quality index (WQI) as well as the hazard quotient (HQ) and hazard index (HI). This paper is aiming to help develop local water management strategies for preventing hazardous contamination.

## 2. Materials and Methods

### 2.1. Regional Geography

**Figure 1:**
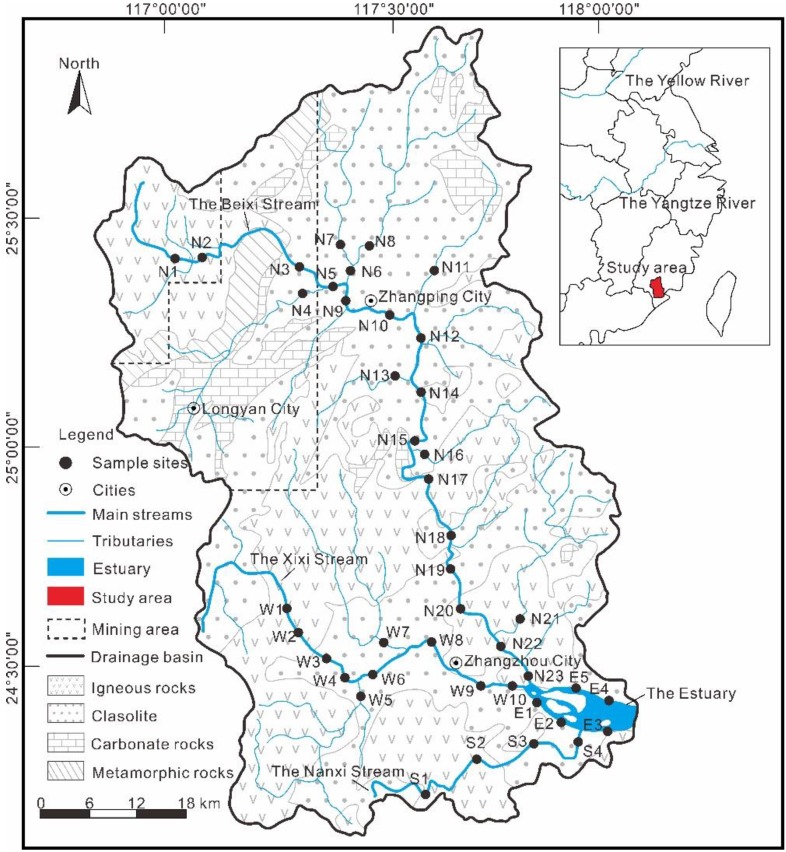
Regional lithology, mining distribution and sampling sites in the Jiulongjiang River.

The Jiulongjiang River is located in the south of Fujian Province (24°13′–25°51′ N, 116°47′–118°02′ E) (Figure 1), and consists of the Beixi stream, Xixi stream, Nanxi stream, and the regional estuary. The Beixi stream is the main stream in the drainage basin, and the Xixi stream and Nanxi stream flow towards the estuary. The drainage basin has an area of 14,700 km^2^ [35], and the length of the river is approximately 1723 km, with an average annual runoff of 1.4 × 10^10^ km^3^ [33,36]. The study area is situated in the subtropical oceanic monsoon climate zone, with an average annual temperature around 21 °C and annual precipitation of 1200–2000 mm [31,33].

The drainage basin is an important area where the Eurasian plate interacted with the Pacific plate [37]. Multi-stage magmatic activities took place in the Mesozoic, and the medium-acidic magmatic rocks were widely distributed. Intrusive rocks and volcanic rocks are mainly distributed in the central and southern parts of the basin, accounting for about two-thirds of the total drainage area [33]. Sedimentary rocks and coal-bearing interlayers are exposed in the northeastern part of the drainage basin and the source of the Xixi stream. The downstream of the Xixi stream deposits a series of alluvial stratum. Carbonates are distributed in the upper reaches of the Beixi stream [38].

### 2.2. Sample Collection and Preparation

Sampling was conducted from downstream to upstream of each stream on the basis of hydrological conditions, lithology, and land use. Water samples were collected from 42 sites along the Beixi stream, Xixi stream, Nanxi stream, and estuary of the Jiulongjiang River, labeled N1–23, W1–10, S1–4, and E1–5 (Figure 1), respectively, during 24–29 July 2017. Before collection, all instruments were rinsed three times with river water. Samples were collected using a water sampler and stored in pre-cleaned high-density polyethylene (HDPE) plastic bottles. For subsequent determination of dissolved heavy metals, water samples were filtered through a 0.22-μm filter membrane on site. Then, water temperature and pH were immediately determined using a YSI water quality monitoring meter (Xylem Inc., Yellow Springs, OH, USA). Finally, the filtrate was acidified to pH ≤ 2 using ultra-pure concentrated nitric acid and stored at approximately 4 °C before element determination.

### 2.3. Dissolved Heavy Metals Determination

The dissolved heavy metals, including Ti, Cr, Mn, Fe, Ni, Mo, Sb, and Ba, were analyzed using inductively coupled plasma-mass spectrometry (ICP-MS, Elan 9000, Perkin Elmer Optima, Waltham, MA, USA) in Tianjin Key Laboratory of Water Resources and Environment, China. The uncertainty of the standard reference material (GSB 04-1767-2004) was below 0.7%. Procedural blanks were determined between every 10 water samples to control the accuracy. Method precision was controlled by re-determining randomly replicate samples between every 10 water samples. Good agreement was obtained between the determined concentrations and standard reference material. The relative standard deviations (RSDs) of elements were below 6.1%. For comparing the distribution and exploring the possible sources of dissolved heavy metals in this study, all of the data were processed using Microsoft Office 2019 (Microsoft Corporation, Redmond, Seattle, WA, USA) and SPSS 19.0 (IBM Corporation, Armonk, NY, USA).

### 2.4. Water Quality Index

The WQI has been emerging as a powerful tool to assess water quality. The impacts caused by different water quality variables were calculated with the following equation:

WQI = Σ [W_i_ × (C_i_/S_i_)] × 100, (1)

where W_i_ represents the weight of each parameter and is calculated on the basis of the eigenvalues for each principal component and factor loading for each parameter from the FA/PCA results [29,39]; C_i_ represents the concentrations of each dissolved heavy metal; and S_i_ represents the guideline values of Chinese drinking water (GB 5749-2006) for each element. The WQI values were classified into five categories: (i) excellent water quality (0 ≤ WQI < 50); (ii) good water quality (50 ≤ WQI < 100); (iii) poor water quality (100 ≤ WQI < 200); (iv) very poor water quality (200 ≤ WQI < 300); and (v) water unsuitable for drinking (WQI ≥ 300) [24].

### 2.5. Health Risk Assessment

Hazard quotient and HI are calculated to evaluate the toxicity of hazardous substances and factors in aqueous systems with quantitative assessments [40,41]. The ingestion of elements through the mouth and nose and absorption through the skin are the three most common exposure pathways [16]. Hazard quotient is the ratio between exposure through individual pathways and the reference dose [42]. Hazard index is the sum of the HQs for individual elements from both the applicable pathways mentioned previously, which is used to analyze the total potential non-carcinogenic risk [43,44]. Non-carcinogenic risk or adverse effects on human health exist when HQ or HI is ≥ 1; no deleterious effects exist when HQ or HI is < 1. Equations to calculate HQ and HI values are as follows:

ADD_ingestion_ = (C_w_ × IR × EF × ED)/(BW × AT), (2)

ADD_dermal_ = (C_w_ × SA × K_p_ × ET × EF × ED × 10^−3^)/(BW × AT), (3)

HQ = ADD/RfD,(4)

RfD_dermal_ = RfD × ABS_GI_, (5)

HI = ΣHQs,(6)

where the meanings and their units in these equations are as follows: ADD_ingestion_ and ADD_dermal_ represents the average daily dose from ingestion and dermal absorption, respectively (μg/kg/day); C_w_ is the average concentration of each element in water (μg/L); BW is the average body weight (70 kg for adults and 15 kg for children); IR is the ingestion rate (2 L/day for adults and 0.64 L/day for children); EF is the exposure frequency (350 days/year); ED is the exposure duration (30 years for adults and 6 years for children); AT is the average time (=ED × 365 days/year); SA represents the exposed skin area (18,000 cm^2^ for adults and 6600 cm^2^ for children); ET is the exposure time (0.58 h/day for adults and 1 h/day for children); K_p_ represents dermal permeability coefficient in water (cm/h); RfD is the reference dose (μg/kg/day); ABS_GI_ is the gastrointestinal absorption factor (dimensionless). The reference values were obtained from the United States Environmental Protection Agency (US EPA) [42].

## 3. Results and Discussion

### 3.1. Water Parameters and Distribution of Dissolved Heavy Metals

The parameters of water samples and concentrations of dissolved heavy metals are listed in Table 1. The Kolmogorov-Smirnov (K-S) test was used to compare a sample with a reference probability distribution, and the results showed that temperature, pH, Ti, and Fe were normally distributed (bilateral significance > 0.100). The coefficient of variation (CV) for the elements indicated that the mean concentration values might have been affected by abnormal interference, resulting in extremely high values. Thus, median values would be better than arithmetic mean values in this study. However, the results reported in the guidelines and other rivers were given as mean values for comparison.

The temperature of water ranged from 21.5 °C to 31.8 °C, with an average of 26.0 °C. Samples collected from Beixi stream had a lower temperature than those from the Nanxi stream and Xixi stream. The pH values varied between weak acidic and weak alkaline (6.42 to 7.60), basically within the guideline (6.5–8.5) reported by GB 5749-2006 [45]. The values of dissolved heavy metals decreased in the following order: Ti > Fe > Ba > Mn > Sb > Mo > Ni > Cr. Based on the average concentrations, the selected elements in water were classified into three categories: (1) Cr, Ni, and Mo (<1 μg/L); (2) Mn and Sb (1–10 μg/L); (3) Ti, Fe, and Ba (>10 μg/L) (Table 1). Cr, Fe, Ni, Mo, and Ba complied with guidelines for drinking water recommended by China (GB 5749-2006), the WHO, and the US EPA, while Ti, Mn, and Sb were not within approved limits at some sites and might be affected by intensive anthropogenic activities along the river. Based on the heavy metals in major world rivers, Ti and Sb were relatively higher, and Ni and Ba were similar to the mean values [46]. Compared with recent published works, the results of Cr, Mn, and Ni were similar to those from the Yangtze River [9] and slightly lower than those from the Pearl River [47]; the concentrations of Ba were similar to those from the Douro River, Portugal [48]; the concentrations of Mo were similar to the water from the Calore River, southern Italy [19]; and the value of Fe in the Jiulongjiang River was similar to that in the Han River [16].

**Figure 2:**
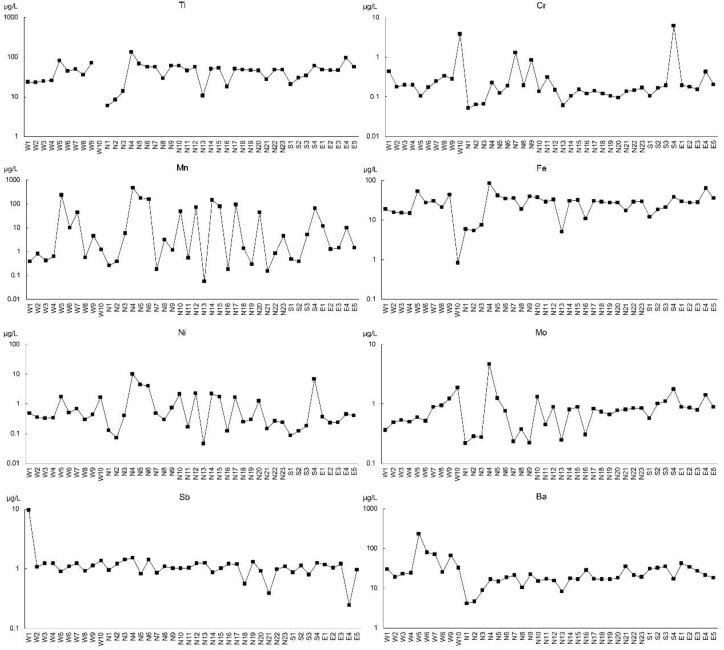
Spatial distributions of dissolved heavy metals in the Jiulongjiang River.

The distribution of each element is individually plotted in Figure 2, and the original data is shown in the Supplementary Materials (Table S1). Based on the distribution characteristics of eight elements, we concluded that the pattern of Ti concentrations were highly similar with that of Fe concentrations in terms of spatial distribution across the whole drainage basin. Ba was slightly similar with Ti and Fe in the Xixi stream. In the Beixi stream, Mn, Ni, and Mo showed a good homogeneity in the upper and middle reaches (N1–17), similar to Ti and Fe. In the Nanxi stream, Ti, Fe, and Mo were distributed in a similar pattern, and Cr, Mn, and Ni were distributed in another pattern with a peak value in the sample from site S4. Sb had a flat pattern, except for sample W1. Overall, the distributions of Ti, Mn, Fe, Ni, and Mo showed a good similarity, indicating that they might be affected by similar natural and anthropogenic activities along the river.

### 3.2. Source Identification of Dissolved Heavy Metals

#### 3.2.1. Correlation Analysis

A Pearson correlation matrix was used to investigate the relations and interactions of dissolved heavy metals in the Jiulongjiang River water [19,25,29]. Strong correlation coefficients might result from similar hydro-geochemical characteristics in the different sites of the study area [51]. The results are shown in Table 2. Strong positive correlation coefficients were observed ranging from 0.630 to 0.996 between each pair of Ti, Mn, Fe, Ni, and Mo, at the 0.01 level, where Ti and Fe were highly correlated, corresponding to the homogeneous spatial distribution of these two elements in Figure 2. Moreover, Mn, Ni, and Mo add might be distributed throughout the Jiulongjiang River in a similar way with Ti and Fe. Cr and Ni had a strong positive correlation coefficient of 0.393 at the 0.05 level, indicating similar sources of Cr and Ni. Ba had a low correlation with other elements. The strong correlations at different levels among the metals indicated multiple sources could be responsible for the dissolved heavy metals present in water of the Jiulongjiang River.

#### 3.2.2. Factor and Principal Component Analysis

**Figure 3:**
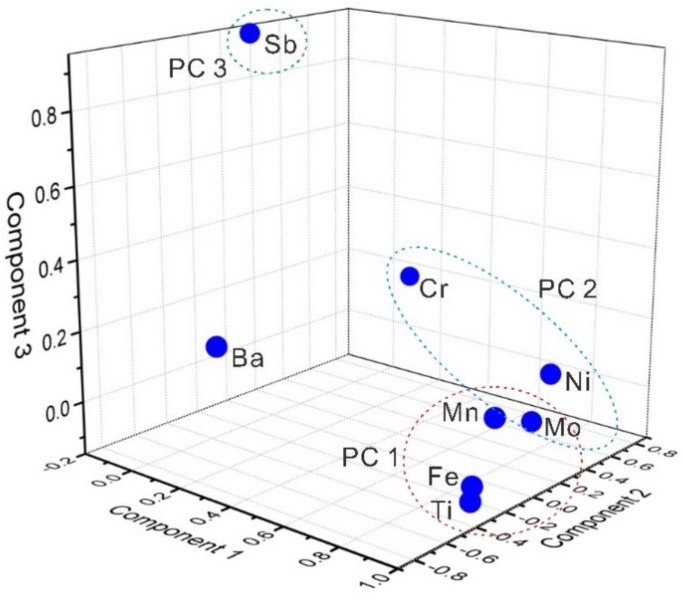
Loading plot of factors or dissolved heavy metals in the Jiulongjiang River.

Factor and Principal Component Analysis is a statistical method to explain the underlying structure of a set of variables and interpret the relationships between variables by converting a large number of potentially related variables into a set of linearly unrelated variables using a dimensionality reduction technique [25,52]. The data needed to avoid numerical ranges of the original variables by z-scale standardization [53]. The reliability of the data for FA/PCA was under 0.001, as calculated by Kaiser-Meyer-Olkin and Bartlett’s sphericity test. The results of FA/PCA for metal concentrations are listed in Table 3. In order to identify the sources of the dissolved heavy metals, the factor scores for each element are plotted in Figure 3.

Three principal components (PC) with eigenvalues were extracted accounting for 79.46% of the total variance. The factor loadings were classified into three groups: strong, moderate, and weak, with the loading values of >0.75, 0.75–0.50, and 0.50–0.30 [52], respectively. PC 1 had strong loadings of Ti (0.90), Mn (0.90), Fe (0.90), Ni (0.88), and Mo (0.85), with a variance of 50.03% (Figure 1), indicating a mixed source of weathering rocks and urban land. Ti and Fe, which are the major elements in crustal materials, were major components with relatively high values [27,54], and the low concentrations of Mn, Ni, and Mo were also representative of their background levels. Therefore, a lithogenic origin might be presumed, since these heavy metals are generally present in weathered rocks [41,55]. Moreover, Ti, Mn, Fe, Ni, and Mo were enriched in site W5 and N4, as well as their nearby area, which were defined as urban land [31], indicating distinct urban land pollution. PC 2, responsible for 16.88% of the variance, was correlated with Cr (0.71), Ni (0.37), and Mo (0.30), which are associated with the discharge of industrial effluents from the factories. It has been reported that some factories are located in the estuary wharf [52,56]. PC 3 had a strong positive loading of Sb (0.95), with a variance of 12.56%. Mine deposits and coal areas are distributed in the upper reaches of Beixi stream (Figure 1) [32], which might be the main factor to cause high loading of Sb in the drainage basin [57,58].

### 3.3. Water Quality Index and Health Risk Assessment

#### 3.3.1. Water Quality Index

**Figure 4:**
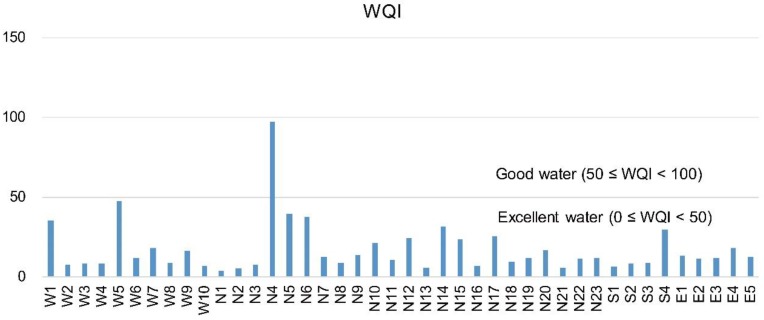
Water quality index (WQI) values of water in the Jiulongjiang River.

The weights of each parameter for calculating the WQI were obtained on the basis of the FA/PCA, and the weights are summarized in Table 4. The values of WQI were calculated with Equation (1), and the WQI results of each sampling site are shown in Figure 4 and in the Supplementary Materials (Table S1). The WQI values varied from 4.23 to 97.65, among which water samples at site N4 (WQI = 97.65) were categorized as good water, and water samples at W5 had a value near the good water limit (WQI = 47.33). In these two sites, water might have been polluted by anthropogenic activities. Water in other sites of this study area fell into the category of excellent water, with WQI values less than 50, indicating the natural water was suitable for drinking. In general, the water quality of Jiulongjiang River was good, but more attention should be paid to sites N4 and W5. On the basis of source identification analyzed by multivariate statistics, Ti, Mn, Fe, and Ni were controlled factors in these two sites, and thus, our study results suggest that the local government should introduce policies to control urban land pollution and industrial emissions.

#### 3.3.2. Health Risk Assessment

**Figure 5:**
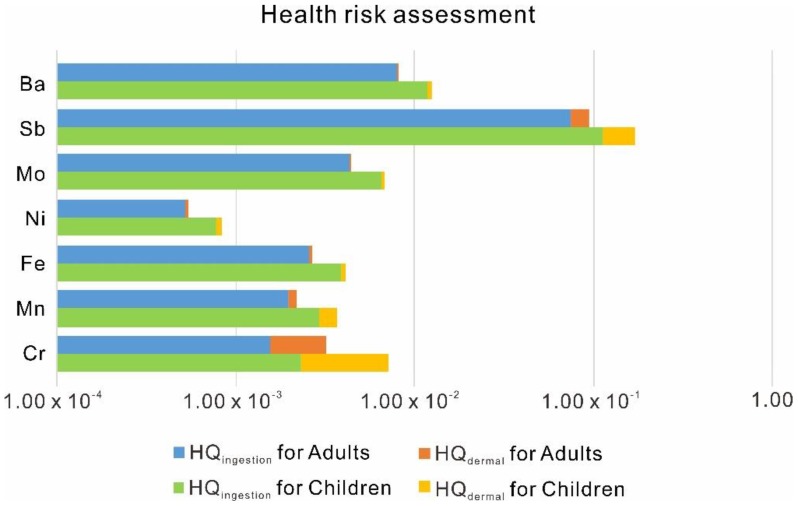
Hazard quotient and hazard index for each element of the Jiulongjiang River.

Based on the risk assessment guidance [42], HQ and HI values for Cr, Mn, Fe, Ni, Mo, Sb, and Ba by ingestion and dermal pathways for adults and children are shown in Figure 5, and the calculation processes are provided in Supplementary Materials (Tables S2–S6), where the HI values were the sum of HQ_ingestion_ and HQ_dermal_. The missing values of Ti were caused by the lack of RfD_ingestion_ and RfD_dermal_ values. For adults, the HQ_ingestion_ and HQ_dermal_ for all elements were smaller than 1, indicating these elements presented little hazard through ingestion and dermal absorption in the whole drainage area. Comparatively, the HQ_ingestion_ and HQ_dermal_ for children were higher, implying children were more sensitive to the dissolved heavy metals in the water. A health risk assessment showed that the HI values of Sb were closer to 1 (0.09 for adults and 0.17 for children) than the other elements, indicating that Sb might represent a potential threat to the water quality of the river. Therefore, we also suggest that more attention should be paid towards controlling mining activities in order to decrease the levels of Sb inputted into the Jiulongjiang River.

## 4. Conclusions

This study presented data of dissolved heavy metals (Ti, Cr, Mn, Fe, Ni, Mo, Sb, and Ba) in the Jiulongjiang River water in order to analyze the distribution and sources of pollution in the water, by using multivariate statistical methods, including CA and FA/PCA. We also provided important information that is relevant to local water management officials by assessing water quality and health risk. Our results arrive at the following conclusions:

(i) Three groups of dissolved heavy metals were categorized: (1) Cr, Ni, and Mo (<1 μg/L); (2) Mn and Sb (1–10 μg/L); (3) Ti, Fe, and Ba (>10 μg/L), in which Ti, Mn, Sb were not within accepted limits at some sites on the basis of guidelines established by the WHO, the US EPA, and China, indicating that they might be the main pollutants of concern in the drainage basin.
(ii) Based on the spatial distributions and CA results, Ti, Mn, Fe, Ni, and Mo all had a high correlation coefficient, indicating a similar source. The FA/PCA results identified three components that accounted for 79.46% of the total variance. PC 1 suggested that a mixed lithogenic and urban land source contributed to Ti, Mn, Fe, Ni, and Mo; PC 2 explained that Cr, Ni, and Mo were also influenced by the discharge of industrial effluents; Sb had a strong loading on PC 3, which was controlled by mining activities.
(iii) The results of WQI indicated that the water in the Jiulongjiang River was basically categorized as excellent water, and water in sites W5 and N4 were poorer because of urban land use. HQ and HI values indicated that Sb was a potential threat to human health. Mining activities in the upper reaches of Beixi stream should be closely scrutinized, with preventive actions taken, if needed.

## Figures and Tables

**Table 1:** Physicochemical parameters and concentrations of dissolved heavy metals in the Jiulongjiang River and from other places.

	Min	Max	Median	Mean	SD ^1^	CV ^2^	K-S Test ^3^	Guidelines for Drinking Water			World	Douro	Calore	Le’an	Pearl	Yangtze	Han
								China ^5^	WHO ^6^	US PEA ^7^	River ^8^	River ^9^	River ^10^	River ^11^	River ^12^	River ^13^	River ^14^
**Water parameters**																	
T (°C)	21.5	31.8	26.0	26.5	2.17	0.08	0.452										
pH	6.42	7.60	7.19	7.16	0.27	0.04	0.985	6.50–8.50									
**Dissolved heavy metals (μg/L)**																	
Ti	nd ^4^	135.03	47.34	44.39	24.76	0.56	0.487	100			3			2.69			
Cr	0.05	6.25	0.17	0.45	1.08	2.41	0	50	50	100	1	2.12	19.7		1.70	0.26	8.14
Mn	0.06	473.70	1.42	40.25	86.59	2.15	0	100	400		7		1035		1.06	2.53	30.72
Fe	0.83	86.03	28.18	27.47	15.73	0.57	0.653	300	300		40					12.53	30.64
Ni	0.05	10.05	0.38	1.17	1.93	1.65	0.001	20	70		0.3	5.93	24.6		1.89	0.18	1.71
Mo	0.22	4.60	0.79	0.85	0.71	0.83	0.015	70			6	15.09	0.89				
Sb	0.25	9.67	1.09	1.26	1.34	1.06	0	5	20	6	0.07	1.98	0.33				41.58
Ba	4.14	231.42	20.12	29.64	35.20	1.19	0	700	1300	2000	20	19.24	194	44.55			87.47

^1^ SD: standard deviation; ^2^ CV: the coefficient of variation; ^3^ K-S test: the Kolmogorov-Smirnov (K-S) test; ^4^ nd: no data. Data sources: ^5^ Chinese Ministry of Health [45]; ^6^ WHO (the World Health Organization) [49]; ^7^ US EPA (the United States Environmental Protection Agency) [50]; ^8^ Li [46]; ^9^ Ribeiro et al. [48]; ^10^ Zuzolo et al. [19]; ^11^ Jiang et al. [10]; ^12^ Wang [47]; ^13^ Li et al. [9]; ^14^ Li and Zhang [16].

**Table 2:** Pearson correlation matrix of dissolved heavy metals in the Jiulongjiang River.

	Ti	Cr	Mn	Fe	Ni	Mo	Sb	Ba
Ti	1							
Cr	−0.014	1						
Mn	0.698 **	−0.018	1					
Fe	0.996 **	0.010	0.696 **	1				
Ni	0.630 **	0.393 *	0.857 **	0.632 **	1			
Mo	0.649 **	0.266	0.726 **	0.653 **	0.802 **	1		
Sb	−0.151	0.032	−0.029	−0.111	0.003	−0.073	1	
Ba	0.248	−0.047	0.243	0.257	−0.036	−0.034	−0.016	1

*: strong positive correlation coefficients at the 0.05 level; **: strong positive correlation coefficients at the 0.01 level.

**Table 3:** Varimax rotated component matrix of dissolved heavy metals in the Jiulongjiang River.

Variables	PC 1	PC 2	PC 3	Communalities
Ti	0.90	−0.24	−0.14	0.88
Cr	0.17	0.71	0.20	0.58
Mn	0.90	−0.08	0.06	0.81
Fe	0.90	−0.23	−0.10	0.87
Ni	0.88	0.37	0.10	0.92
Mo	0.85	0.30	−0.03	0.82
Sb	−0.08	0.01	0.95	0.91
Ba	0.22	−0.69	0.21	0.57
Eigenvalues (%)	4.00	1.35	1.00	
Variance (%)	50.03	16.88	12.56	
Cumulative (%)	50.03	66.90	79.46	

PC: principal component. Significance of Kaiser-Meyer-Olkin and Bartlett’s sphericity test is <0.001. Extraction method: Principal component analysis. Rotation method: Varimax with Kaiser normalization. Rotation converges after four iterations.

**Table 4:** Weights for the variables in the water samples from the Jiulongjiang River.

PC	Eigenvalue (%)	Relative Eigenvalue	Variable	Loading Value	Relative Loading Value on Same PC	Weight ^1^
1	4.00	0.63	Ti	0.90	0.20	0.13
			Mn	0.90	0.20	0.13
			Fe	0.90	0.20	0.13
			Ni	0.88	0.20	0.13
			Mo	0.85	0.19	0.12
			Total	4.43	1.00	0.63
2	1.35	0.21	Cr	0.71	0.51	0.11
			Ni	0.37	0.27	0.06
			Mo	0.30	0.22	0.05
			Total	1.38	1.00	0.21
3	1.00	0.16	Sb	0.95	1.00	0.16
			Total	0.95	1.00	0.16
Total	6.36					1.00

^1^ Weight = relative eigenvalue × relative loading value.

## Supplementary Materials

The following are available online at http://www.mdpi.com/1660-4601/15/12/2752/s1, Table S1. Original data and water quality index (WQI) results of dissolved heavy metals in the Jiulongjiang River. Table S2. Hazard quotient by ingestion pathway of dissolved heavy metals for adults. Table S3. Hazard quotient by ingestion pathway of dissolved heavy metals for children. Table S4. Hazard quotient by dermal pathway of dissolved heavy metals for adults. Table S5. Hazard quotient by dermal pathway of dissolved heavy metals for children. Table S6. Hazard index of dissolved heavy metals in the Jiulongjiang River.
