# Supplementary material for: Distribution, Sources, and Water Quality Assessment of Dissolved Heavy Metals in the Jiulongjiang River Water, Southeast China
Source: Int J Environ Res Public Health. 2018 Dec 5;15(12):2752. doi: 10.3390/ijerph15122752 (PMC6314042; doi:10.3390/ijerph15122752)
Supplement: Supplementary file 1 [file ijerph-15-02752-s001.pdf]

Table S1. Original data and water quality index (WQI) results of dissolved heavy metals in the Jiulongjiang River. (concentration unit of the elements: μg/L)

	T (°C)	pH	Ti	Cr	Mn	Fe	Ni	Mo	Sb	Ba	WQI
W1	24.3	7.18	23.79	0.44	0.39	18.80	0.48	0.36	9.67	29.62	13.13
W2	25.1	7.31	22.74	0.18	0.82	15.80	0.35	0.49	1.07	19.13	5.68
W3	25	7.38	24.63	0.20	0.41	15.06	0.33	0.53	1.23	22.81	6.05
W4	25.5	7.4	25.83	0.20	0.62	14.78	0.34	0.50	1.24	24.43	6.27
W5	28	6.71	81.95	0.11	229.26	52.43	1.73	0.59	0.90	231.42	50.67
W6		7.13	44.34	0.18	9.78	27.19	0.50	0.51	1.09	79.56	11.76
W7	26.4	6.87	48.85	0.24	43.50	30.57	0.69	0.89	1.23	71.93	17.13
W8	27.3	7.04	36.36	0.34	0.57	21.05	0.29	0.95	0.91	25.30	7.86
W9	28	6.94	71.53	0.28	4.46	43.95	0.44	1.22	1.14	66.76	15.52
W10	28.1	7.01		3.85	1.19	0.83	1.64	1.86	1.38	32.16	4.35
N1	21.5	7.41	5.99	0.05	0.26	5.86	0.13	0.22	0.94	4.14	2.14
N2	23.8	6.44	8.39	0.06	0.38	5.38	0.07	0.28	1.21	4.65	2.65
N3	23.6	6.42	13.99	0.07	5.77	7.46	0.40	0.27	1.42	8.93	4.77
N4	25.4	6.81	135.03	0.23	473.70	86.03	10.05	4.69	1.51	16.55	95.43
N5	24.5	6.85	67.66	0.12	170.15	41.54	4.53	1.25	0.82	14.56	38.57
N6	24.9	6.95	55.77	0.19	157.50	34.18	3.95	0.76	1.42	18.67	34.91
N7	25.4	7.2	55.81	1.30	0.19	35.42	0.48	0.23	0.85	21.01	11.27
N8	24.7	7.4	29.06	0.19	3.20	18.66	0.30	0.38	1.10	10.51	6.79
N9	26.2	7.6	61.24	0.84	1.14	39.49	0.74	0.22	1.02	22.63	12.65
N10	25	6.97	61.69	0.14	47.89	37.08	2.10	1.31	1.01	14.99	19.74
N11	25.3	7.27	45.53	0.31	0.54	28.66	0.17	0.44	1.03	17.28	9.23
N12	24.9	7.09	55.93	0.15	70.92	33.14	2.25	0.89	1.23	15.56	21.98
N13	25.5	7.46	10.59	0.06	0.06	5.04	0.05	0.25	1.25	8.15	2.98
N14	24.8	6.95	51.07	0.11	146.37	29.96	2.23	0.80	0.86	17.56	30.67
N15	25.8	7.46	53.61	0.15	79.38	31.62	1.71	0.88	1.01	16.65	22.07
N16	25.7	7.55	18.00	0.12	0.18	10.85	0.12	0.30	1.21	28.12	4.76
N17	25.7	7.23	50.77	0.14	92.35	30.37	1.67	0.82	1.19	17.34	23.42
N18	26.8	7.38	47.67	0.12	1.36	28.45	0.25	0.74	0.56	16.69	9.32
N19	26.3	7.27	46.52	0.10	0.30	27.42	0.30	0.66	1.29	16.68	9.58
N20	27.2	7.34	46.06	0.09	44.62	27.07	1.26	0.78	0.91	18.20	15.83
N21	31.8	7.6	27.96	0.14	0.15	17.35	0.15	0.80	0.39	34.63	6.03
N22	26.8	7.08	48.63	0.14	0.83	28.73	0.26	0.84	0.97	21.35	9.86
N23	29.3	7.15	48.48	0.17	4.51	29.20	0.24	0.85	1.10	19.23	10.38
S1	29.3	7.43	20.51	0.11	0.49	12.11	0.09	0.58	0.87	30.40	4.99
S2	30.2	7.00	29.91	0.16	0.39	18.43	0.12	1.01	1.12	32.57	6.95
S3	31.7	7.00	33.99	0.20	5.09	21.10	0.18	1.10	0.80	34.96	8.12
S4	31.1	7.37	60.26	6.25	65.85	38.72	6.71	1.76	1.26	17.28	27.29
E1	27.3	7.11	47.92	0.19	11.87	29.38	0.37	0.89	1.17	42.09	11.90
E2	27.6	7.19	47.28	0.18	1.23	27.57	0.23	0.87	1.03	33.91	9.94
E3	27.6	7.15	47.41	0.15	1.41	27.91	0.24	0.79	1.22	26.87	10.00
E4	28.5	7.31	95.58	0.43	10.00	63.49	0.45	1.41	0.25	21.43	18.97
E5	27.4	7.2	56.15	0.20	1.44	35.75	0.41	0.89	0.96	17.97	11.38

Table S2. Hazard quotient by ingestion pathway of dissolved heavy metals for adults.

**Adults**	**C_w**	**IR**	**EF**	**ED**	**BW**	**AT**	**$ADD_{ingestion}$**	**$RfD_{ingestion}$**	**$HQ_{ingestion}$**
Cr	0.17	2	350	30	70	10950	4.66×10^{-03}	3	1.55×10^{-03}
Mn	1.42	2	350	30	70	10950	3.89×10^{-02}	20	1.95×10^{-03}
Fe	28.18	2	350	30	70	10950	7.72×10^{-01}	300	2.57×10^{-03}
Ni	0.38	2	350	30	70	10950	1.04×10^{-02}	20	5.21×10^{-04}
Mo	0.79	2	350	30	70	10950	2.16×10^{-02}	5	4.33×10^{-03}
Sb	1.09	2	350	30	70	10950	2.99×10^{-02}	0.4	7.47×10^{-02}
Ba	20.12	2	350	30	70	10950	5.51×10^{-01}	70	7.87×10^{-03}

Table S3. Hazard quotient by ingestion pathway of dissolved heavy metals for children.

**Children**	**C_w**	**IR**	**EF**	**ED**	**BW**	**AT**	**$ADD_{ingestion}$**	**$RfD_{ingestion}$**	**$HQ_{ingestion}$**
Cr	0.17	0.64	350	6	15	2190	6.96×10^{-03}	3	2.32×10^{-03}
Mn	1.42	0.64	350	6	15	2190	5.81×10^{-02}	20	2.90×10^{-03}
Fe	28.18	0.64	350	6	15	2190	1.15	300	3.84×10^{-03}
Ni	0.38	0.64	350	6	15	2190	1.55×10^{-02}	20	7.77×10^{-04}
Mo	0.79	0.64	350	6	15	2190	3.23×10^{-02}	5	6.46×10^{-03}
Sb	1.09	0.64	350	6	15	2190	4.46×10^{-02}	0.4	1.11×10^{-01}
Ba	20.12	0.64	350	6	15	2190	8.23×10^{-01}	70	1.18×10^{-02}

Table S4. Hazard quotient by dermal pathway of dissolved heavy metals for adults.

**Adults**	**C_w**	**SA**	**K_p**	**ET**	**EF**	**ED**		**BW**	**AT**	**ADD_{dermal}**	**RfD_{dermal}**	**HQ_{dermal}**
Cr	0.17	18000	0.001	0.58	350	30	0.001	70	10950	2.43×10^{-05}	0.015	1.62×10^{-03}
Mn	1.42	18000	0.001	0.58	350	30	0.001	70	10950	2.03×10^{-04}	0.8	2.54×10^{-04}
Fe	28.18	18000	0.001	0.58	350	30	0.001	70	10950	4.03×10^{-03}	45	8.96×10^{-05}
Ni	0.38	18000	0.002	0.58	350	30	0.001	70	10950	1.09×10^{-04}	5.4	2.01×10^{-05}
Mo	0.79	18000	0.00166667	0.58	350	30	0.001	70	10950	1.88×10^{-04}	1.9	9.91×10^{-05}
Sb	1.09	18000	0.001	0.58	350	30	0.001	70	10950	1.56×10^{-04}	0.008	1.95×10^{-02}
Ba	20.12	18000	0.001	0.58	350	30	0.001	70	10950	2.88×10^{-03}	14	2.06×10^{-04}

Table S5. Hazard quotient by dermal pathway of dissolved heavy metals for children.

**Children**	**C_w**	**SA**	**K_p**	**ET**	**EF**	**ED**		**BW**	**AT**	**ADD_{dermal}**	**RfD_{dermal}**	**HQ_{dermal}**
Cr	0.17	6600	0.001	1	350	6	0.001	15	2190	7.17×10^{-05}	0.015	4.78×10^{-03}
Mn	1.42	6600	0.001	1	350	6	0.001	15	2190	5.99×10^{-04}	0.8	7.49×10^{-04}
Fe	28.18	6600	0.001	1	350	6	0.001	15	2190	1.19×10^{-02}	45	2.64×10^{-04}
Ni	0.38	6600	0.002	1	350	6	0.001	15	2190	3.21×10^{-04}	5.4	5.94×10^{-05}
Mo	0.79	6600	0.001667	1	350	6	0.001	15	2190	5.56×10^{-04}	1.9	2.92×10^{-04}
Sb	1.09	6600	0.001	1	350	6	0.001	15	2190	4.60×10^{-04}	0.008	5.75×10^{-02}
Ba	20.12	6600	0.001	1	350	6	0.001	15	2190	8.49×10^{-03}	14	6.06×10^{-04}

Table S6. Hazard index of dissolved heavy metals in the Jiulongjiang River.

Element	$HQ_{ingestion}$		HQ_{dermal}		HI	
	Adults	Children	Adults	Children	Adults	Children
Cr	1.55×10^{-03}	2.32×10^{-03}	1.62×10^{-03}	4.78×10^{-03}	3.17×10^{-03}	7.10×10^{-03}
Mn	1.95×10^{-03}	2.90×10^{-03}	2.54×10^{-04}	7.49×10^{-04}	2.20×10^{-03}	3.65×10^{-03}
Fe	2.57×10^{-03}	3.84×10^{-03}	8.96×10^{-05}	2.64×10^{-04}	2.66×10^{-03}	4.11×10^{-03}
Ni	5.21×10^{-04}	7.77×10^{-04}	2.01×10^{-05}	5.94×10^{-05}	5.41×10^{-04}	8.37×10^{-04}
Mo	4.33×10^{-03}	6.46×10^{-03}	9.91×10^{-05}	2.92×10^{-04}	4.43×10^{-03}	6.76×10^{-03}
Sb	7.47×10^{-02}	1.11×10^{-01}	1.95×10^{-02}	5.75×10^{-02}	9.41×10^{-02}	1.69×10^{-01}
Ba	7.87×10^{-03}	1.18×10^{-02}	2.06×10^{-04}	6.06×10^{-04}	8.08×10^{-03}	1.24×10^{-02}

Acronyms: $ADD_{ingestion}$ and ADD_{dermal}: the average daily dose from ingestion and dermal absorption (μg/kg/day); C_w: the average concentration of each element in water (μg/L); BW: the average body weight (70 kg for adults and 15 kg for children); IR: the ingestion rate (2 L/day for adults and 0.64 L/day for children); EF: the exposure frequency (350 days/year); ED: the exposure duration (30 years for adults and 6 years for children); AT: the average time (= ED × 365 days/year); SA: the exposed skin area (18,000 cm^2 for adults and 6600 cm^2 for children); ET: the exposure time (0.58 h/day for adults and 1 h/day for children); K_P: dermal permeability coefficient in water (cm/h); RfD: the corresponding reference dose (μg/kg/day); ABS_{GI}: the gastrointestinal absorption factor
